# Reliability and Repeatability of Quantitative Tractography Methods for Mapping Structural White Matter Connectivity in Preterm and Term Infants at Term-Equivalent Age

**DOI:** 10.1371/journal.pone.0085807

**Published:** 2014-01-24

**Authors:** Supreet Kaur, Samuel Powell, Lili He, Christopher R. Pierson, Nehal A. Parikh

**Affiliations:** 1 Center for Perinatal Research, The Research Institute at Nationwide Children's Hospital, Columbus, Ohio, United States of America; 2 Departments of Pediatrics and Pathology, The Ohio State University College of Medicine, Columbus, Ohio, United States of America; 3 Department of Pathology and Laboratory Medicine, Nationwide Children's Hospital, Columbus, Ohio, United States of America; Beijing Normal University, China

## Abstract

Premature infants exhibit widespread insults and delays in white matter maturation that can be sensitively detected early using diffusion tensor imaging. Diffusion tensor tractography facilitates in vivo visualization of white matter tracts and has the potential to be more sensitive than simpler two-dimensional DTI-based measures. However, the reliability and reproducibility of performing tractography for major white matter tracts in preterm infants is not known. The main objective of our study was to develop highly reliable and repeatable methods for ten white matter tracts in extremely low birth weight infants (birth weight ≤1000 g) at term-equivalent age. To demonstrate clinical utility, we also compared fiber microstructural and macrostructural parameters between preterm and healthy term controls. Twenty-nine ELBW infants and a control group of 15 healthy term newborns were studied. A team of researchers experienced in neuroanatomy/neuroimaging established the manual segmentation protocol based on a priori anatomical knowledge and an extensive training period to identify sources of variability. Intra- and inter-rater reliability and repeatability was tested using intra-class correlation coefficient, within-subject standard deviation (SD), repeatability, and Dice similarity index. Our results support our primary goal of developing highly reliable and reproducible comprehensive methods for manual segmentation of 10 white matter tracts in ELBW infants. The within-subject SD was within 1–2% and repeatability within 3–7% of the mean values for all 10 tracts. The intra-rater Dice index was excellent with a range of 0.97 to 0.99, and as expected, the inter-rater Dice index was lower (range: 0.80 to 0.91), but still within a very good reliability range. ELBW infants exhibited fewer fiber numbers and/or abnormal microstructure in a majority of the ten quantified tracts, consistent with injury/delayed development. This protocol could serve as a valuable tool for prompt evaluation of the impact of neuroprotective therapies and as a prognostic biomarker for neurodevelopmental impairments.

## Introduction

Diffusion tensor tractography (DTT), a three-dimensional diffusion tensor imaging (DTI) technique, is now evolving into a potent investigative tool to study early brain development and white matter structural connectivity in vivo. Diffusion parameters such as fractional anisotropy (FA) and diffusion coefficients, such as mean diffusion (MD), axial diffusivity (AD), and radial diffusivity (RD), provide vital insights into the degree of myelination and white matter organization [1]–[3]. The degree of diffusion within the developing human brain is influenced by many critical factors such as relative membrane permeability of water, tissue water content, degree of myelination and the dense packing of axons. Its application in very preterm infants has the potential to enhance our understanding of the encephalopathy of prematurity that is heavily affected by preoligdendrocyte and axonal injury and aberrant white matter development [3], [4].

Recent studies have made significant progress in mapping detailed human adult brain anatomy of white matter tracts and their connectivity using DTT [1], [2], [5]–[23]. Furthermore, tractography results of major white matter fibers obtained from these adult studies were reported to be in agreement with classical definitions based on postmortem studies. To a lesser degree, DTT has also been used to study neonatal brain development and white matter connectivity. Preliminary studies in preterm infants have explored white matter density and fiber maturation in developing brains [24]–[30]. However, there are significant challenges in studying neonatal white matter brain anatomy and connectivity. The developing neonatal brain has very different tissue characteristics compared to adult brains, such as the degree of myelination and water content, resulting in lower FA values in the white matter tracts [30]. Neonatal scans also have lower image contrast due to incomplete myelination, lower signal-to noise ratio resulting from a need for shorter scan times, and lower spatial resolution due to smaller head size [30]. Recent investigators have demonstrated the feasibility of performing white matter tractography in preterm infants [31], [32]. Additional clinical studies suggest that white matter tract development is adversely affected by premature birth [33]–[35] and that measures of tract diffusion and/or length are promising independent predictors of neurodevelopmental impairments [36]–[37]. Yet, only a few tracts have been studied, and tractography methodology has not been sufficiently tested for reliability or repeatability in very preterm infants.

The main objective of our study was to develop highly reliable and reproducible methods for manual segmentation of ten well-delineated white matter tracts in extremely low birth weight (ELBW; BW ≤1000 g) infants at term-equivalent age. The feasibility of tractography of these ten white matter tracts could serve as a prognostic biomarker for neurodevelopmental impairments in preterm infants. A secondary objective was to compare tract-based parameters between healthy term control infants and ELBW infants. The following ten white matter tracts were categorically segmented and classified: (I) *Commissure fiber tract*: corpus callosum (CC); (II) *Projection fibers*: corticospinal fiber tract (CST); (III) *Association tracts*: inferior longitudinal fasciculus (ILF), inferior-fronto occipital (IFO) tract, and the uncinate fasciculus (UNC) bundle; (IV) *Limbic system tracts*: cingulum in the cingulate gyrus (CG) and the fornix (FX) fiber bundle; (V) *Visual cortex tract*: Optic radiations (OR); (VI) *Cerebral peduncles*: middle cerebellar peduncle (MCP) and superior cerebellar peduncle (SCP).

## Materials and Methods

### Ethics Statement

The study was approved by the Institutional Review Board of the University of Texas Health Science Center at Houston and Children's Memorial Hermann Hospital. Written informed consent was acquired from parents of each ELBW and term participant prior to enrollment and participation in the study.

### Subjects

Based on an assessment of image quality and signal abnormalities such as subject motion and geometric distortions, a study population of 29 ELBW infants was randomly chosen from an imaging cohort of 50 ELBW infants. All infants were cared for in the Children's Memorial Hermann Hospital NICU from May 2007 to July 2009. Infants with severe white matter injury or any major congenital anomalies were excluded. A control group of 16 healthy, 1- to 4-day old full-term newborns (37 to 41 weeks appropriate for gestational age) from the well baby nursery were also selected. All infants were appropriate for gestational age and were excluded if they had any history of perinatal distress or complications (see [37], for additional details). One term infant was excluded for severe motion artifacts that interfered with tract segmentation.

### MRI Acquisition

All subjects were transported to the MRI scanner and supervised during scanning by three experienced neonatal personnel – a transport nurse, a research nurse, and a neonatologist. All MRI scans were performed during natural sleep, without sedation, after infants were fed, swaddled, and restrained in a transporter, MedVac Infant Vacuum Splint (CFI Medical Solutions, Fenton, MI). MRI noise was attenuated using Insta-Puffy Silicone Earplugs (E.A.R. Inc, Boulder, CO) and Natus Mini Muffs (Natus Medical Inc, San Carlos, CA). The MRI scans were performed on a 3T scanner (Achieva, Philips Medical Systems, Best, Netherlands), which was equipped with a 32 channel receiver and a gradient system, capable of producing gradient amplitudes of 80mT/m with a slew rate of 200 T/m/s. A head coil of 8-channel phased array was used for data acquisition purpose. The study DTI protocol comprised of a single-shot, spin-echo planar sequence with TR/TE, 6000/61; in plane resolution 1.6×1.6 mm2, field of view (FOV), 180 mm2; 112×112 matrix; and 2-mm continuous slices. 15 directions of diffusion gradients were used with a b value of 800 s/mm2, and another image with no diffusion gradient was obtained (b = 0 s/mm2).

### Image Processing

The DTI data was transferred to a PC with Windows platform and processed using DTI Studio (software developed by H. Jiang and S. Mori, Johns Hopkins University). FSL software (developed by the Analysis Group, FMRIB, Oxford, UK) was used for eddy current correction using the B0 image, which corrects for imaging artifacts and subject motion. Then we aligned the mean diffusion-weighted images using a 12-point affine AIR program in DTI Studio to remove any small bulk motions that occur during scanning. The lead author then inspected all images for possible artifacts due to infant motion or scanner malfunction. The six elements of the diffusion tensor were calculated for each pixel in DTI Studio. The tractography tool in DTI Studio is based on the Fiber Assignment by Continuous Tracking (FACT) method and brute-force approach, which performs the tracking from all the pixels within the brain [10], [38]–[40]. The eigenvector associated with the largest eigenvalue was used to determine fiber orientation. This method required a specific FA threshold and fiber tracking angle for each fiber tract during segmentation. A multiple ROI seed selection approach was utilized for the 3D white matter tract reconstruction based on existing prior anatomical knowledge of the white matter fiber trajectories. In the multi-ROI approach, three types of functional operations – “OR”, “AND” and “NOT” – were used depending upon the fiber tract trajectory. Because we wished to reproduce full tracts, the “CUT” operation, which restricts tracts between two ROIs, was not used. The OR operation was used to select the fibers passing through either specific ROI-A or ROI-B. It was primarily used to define the first ROI for all the white matter tracts. The AND operation was used to restrict or filter the fibers which penetrate or were common to both ROI-A and ROI-B with known prior knowledge, but also included fibers before ROI-A and after ROI-B. Last, the NOT operation was used to remove extraneous fiber projections which did not belong to the actual fiber trajectory of a desired fiber tract bundle. We used color-coded maps, fractional anisotropy, and trace maps to locate landmarks for segmenting the white matter tracts [41].

### Neonatal Tractography Protocol

The primary anatomical references used were the MRI Atlas of Human White Matter and the Atlas of Human Central Nervous System Development [15], [41]–[44]. Initial ROI selections and placements for each tract were selected by the lead author (SK). All landmarks and placements were then verified by a perinatal brain injury and neuroimaging researcher (NAP) and pediatric neuropathologist and brain injury researcher (CRP), each with greater than 10 years of experience. Next, an extensive 5-month period of discovery and training ensued with the goal of segmentation of as many tracts as possible in the neonatal brain. During this period, optimal FA starting and stopping thresholds and tract turning angles were selected with the goal of optimizing detection of fiber tracts (low false negative rate) without resulting in an excessive false positive rate that could increase detection of noise or non-tract of interest voxels if the selected FA threshold was too low. Some detection of false positives was acceptable because these false positive tracts could be readily eliminated by using the “NOT” function. From this discovery and training period, it was determined that 10 tracts could be readily segmented in our initial group of 15 ELBW infants and 15 healthy control infants. Segmentation of these 10 tracts was then repeated by the lead author two more times to improve reliability and repeatability. The second rater (SP) was only given a one-week period of training before he segmented the full dataset.

#### Corpus Callosum (CC) (Fig. 1)

**Figure 1:**
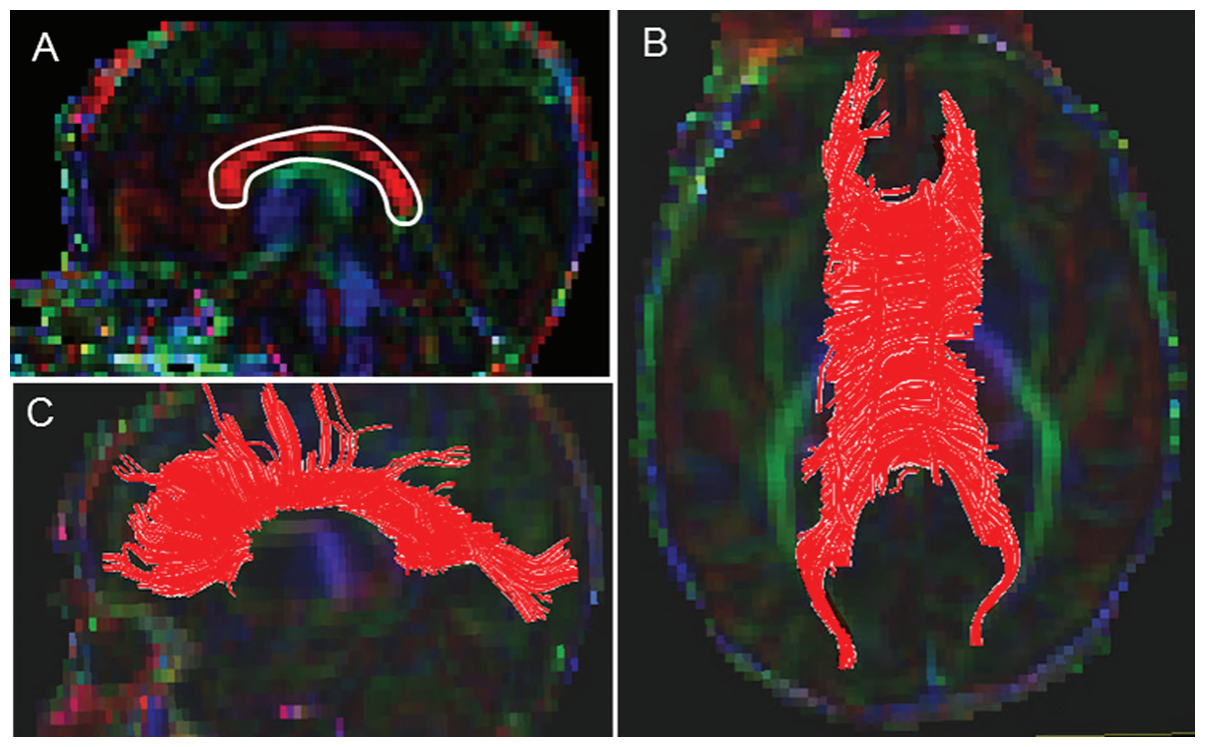
Location of single ROI on DTI color maps for the corpus callosum (CC) in a preterm infant. (A) Polygonal shaped ROI drawn over the CC in sagittal view; (B) Trajectory of the CC tract in axial view; (C) Trajectory of the CC tract in axial view.

A starting FA threshold of 0.10, stopping threshold of 0.10, and turning angle of 70° were selected. For drawing the single ROI, the central sagittal slice in which the body of the corpus callosum appeared to be homogenous in shape was selected (Fig. 1A). Next, the tight polygonal shape tool and the “OR” function in the DTI Studio were selected to delineate the fiber tract on the sagittal image (Fig. 1C). The tight polygonal ROI was drawn around the entire intense red region of the CC fiber bundle, with minimal space remaining between the boundary of this region and the ROI drawing (Fig. 1B). As this approach results in numerous non-CC extraneous fibers, we removed these using the “NOT” function according to our predefined exclusion protocol (see Supplementary Appendix for details). This protocol specified common extraneous fiber tracts that typically are selected using this brute-force approach (e.g. transverse pontine fibers, CST, cingulum) and outlined the order to follow to systematically eliminate them.

#### Corticospinal Tract (CST) (Fig. 2)

**Figure 2:**
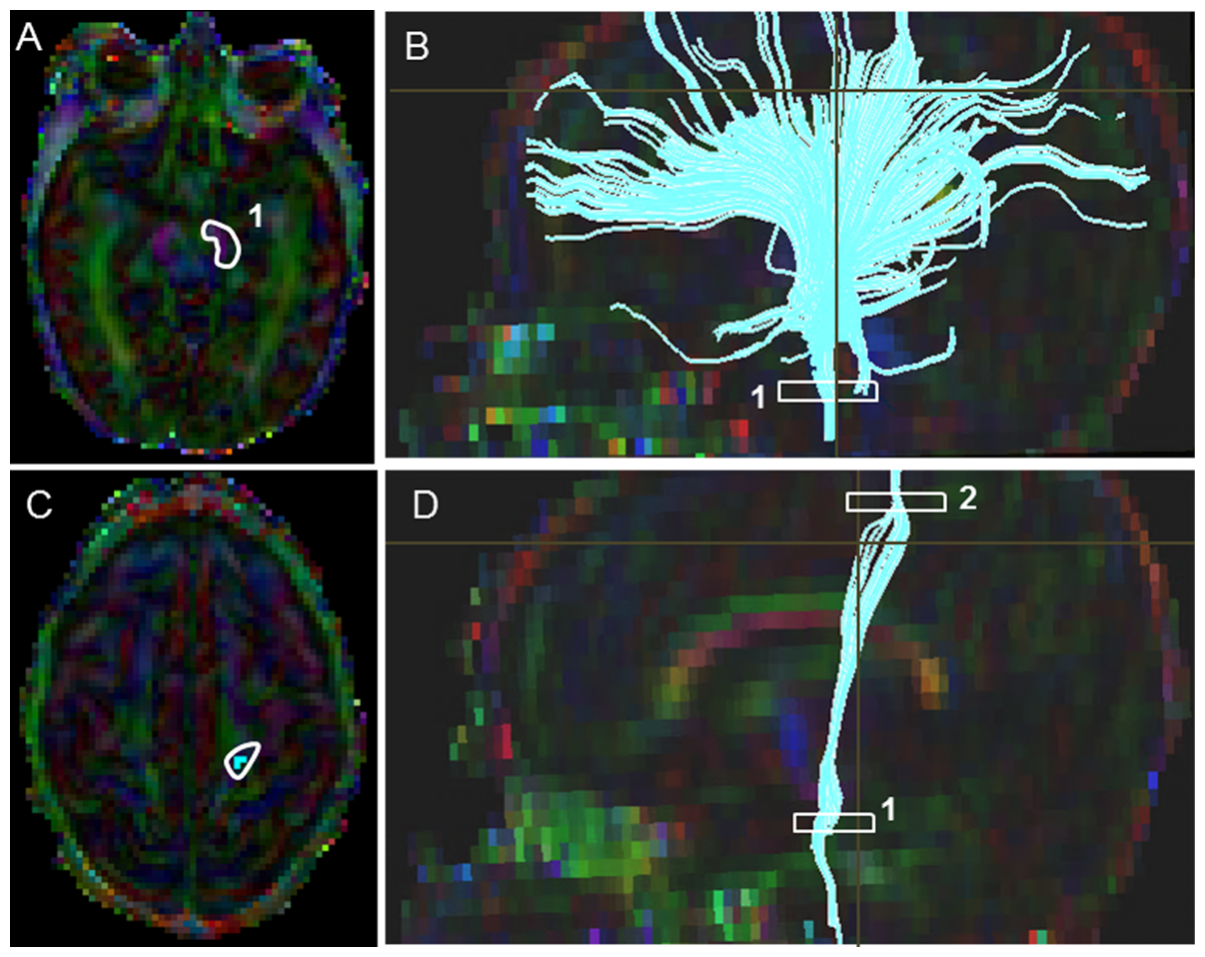
Location of ROIs on DTI color maps for the corticospinal tract (CST) in a preterm infant. (A) Polygonal shaped first ROI drawn over the cerebral peduncle in axial view; (B) Tract after the first ROI was drawn in sagittal view; (C) Second ROI drawn over the pre-central gyrus in axial view; (D) Full trajectory of the CST in sagittal view.

Starting FA threshold of 0.05, the stopping threshold of 0.03, and turning angle of 41° were taken. The first ROI was placed at the level of the decussation of superior cerebellar peduncles (Fig. 2). On the axial Trace image, a heart shaped central structure above the cerebellar region connected by the optic tract above, was located; and then the superior boundary was located using the inter-hemispheric space with a “V”/“M” shaped optic tract as a key landmark. On the color map image, using the “OR” function, a tight poly-shaped ROI around the left purple cerebellar peduncle was drawn (Fig. 2A). Then the second ROI was located using the axial FA map image, the most cephalad (the top most) slice, where the cleavage of the central sulcus was identified. On the color map image using the “AND” function, a tight poly-shaped ROI surrounding precisely the left pre-central gyrus fibers was drawn (Fig. 2C). Last, the non-CST extraneous fibers were removed according to our predefined exclusion protocol that outlined the order to follow to systematically eliminate them (see Supplementary Appendix for details).

#### Inferior Longitudinal Fasciculus (ILF) (Fig. 3)

**Figure 3:**
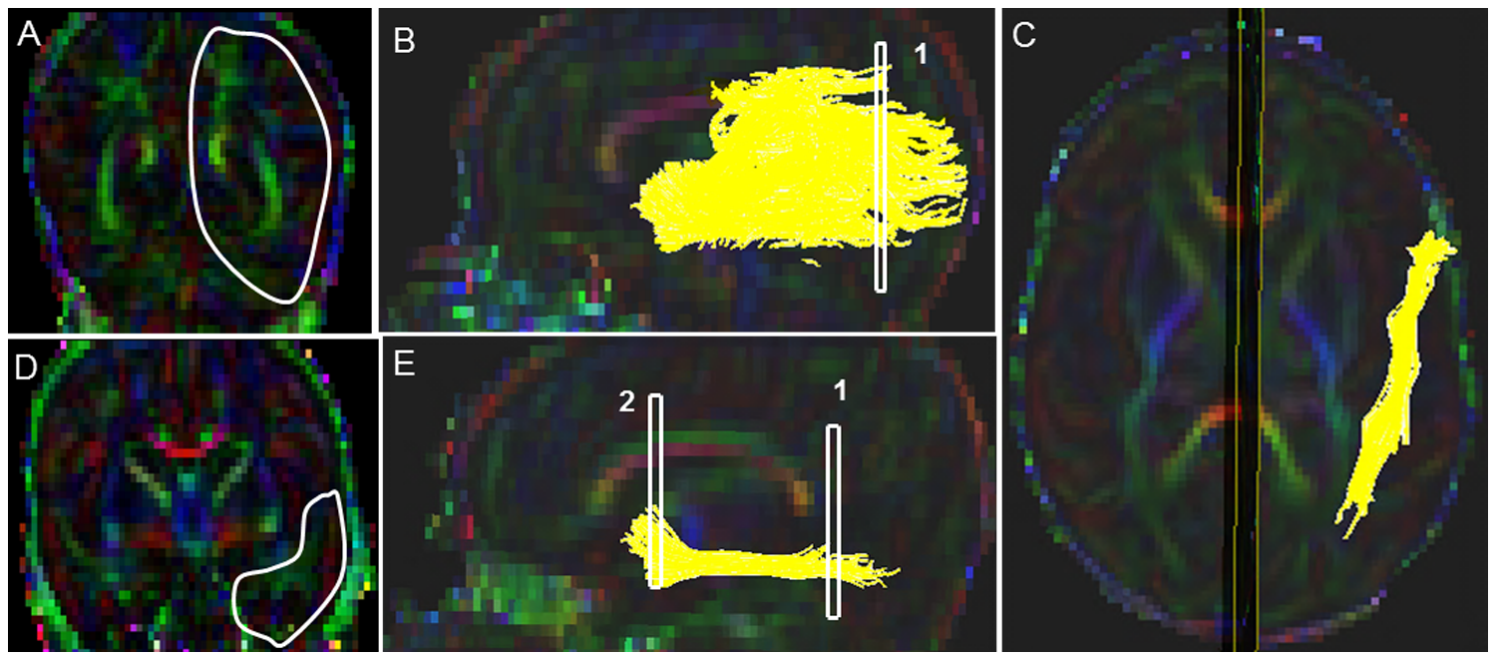
Location of ROIs on DTI color maps for the inferior-longitudinal fasciculus (ILF) in a preterm infant. (A) First ROI for segmenting the ILF tract in coronal view, covering the entire left hemisphere; (B) Fiber trajectory after the first ROI was drawn in sagittal view; (C) Final trajectory of the ILF tract in axial view; (D) Polygonal shaped second ROI in coronal view; (E) Final trajectory of the ILF tract and the locations of the two ROIs in sagittal view.

Starting FA threshold of 0.11 stopping threshold of 0.05, and turning angle of 41° were taken. First the sagittal slice in which cingulum (green fibers) was prominently visible was located in the sagittal view. Then the axial slice marker was placed over the center of the body of the corpus callosum. Following this, the coronal plane was placed in such a manner that it touched the posterior edge of the cingulum (which appeared as green fibers). For the first ROI, a coronal slice was identified at the level of the posterior edge of the intense green cingulum as a prominent landmark, and the coronal slice marker was placed immediately adjacent to the splenium of the corpus callosum (Fig. 3A). The first ROI was drawn using the polygonal shape on the coronal slice, and it delineated the entire cerebral hemisphere. Then second ROI selection was drawn using the “AND function” and polygonal shape on the coronal slice, and was selected in the anterior one-third of the genu of the corpus callosum (as determined by the coronal slice marker on the 2D sagittal slice) (Fig. 3D). This slice was seen in the coronal view as bright intense green cingulum fibers present over the genu of the corpus callosum and intense green posterior limb of the internal capsule, along with intense red color amygdala and purple-colored external capsule. There was a clear anatomical demarcation by the lateral sulcus which separates the frontal lobe from temporal lobe (when seen on the FA and Trace map images). Last, as per our predefined exclusion protocol, non-ILF extraneous (e.g. IFO, CST, cingulum) fibers were systematically removed (see Supplementary Appendix for details).

#### Inferior Fronto-Occipital (IFO) Fasciculus (Fig. 4)

**Figure 4:**
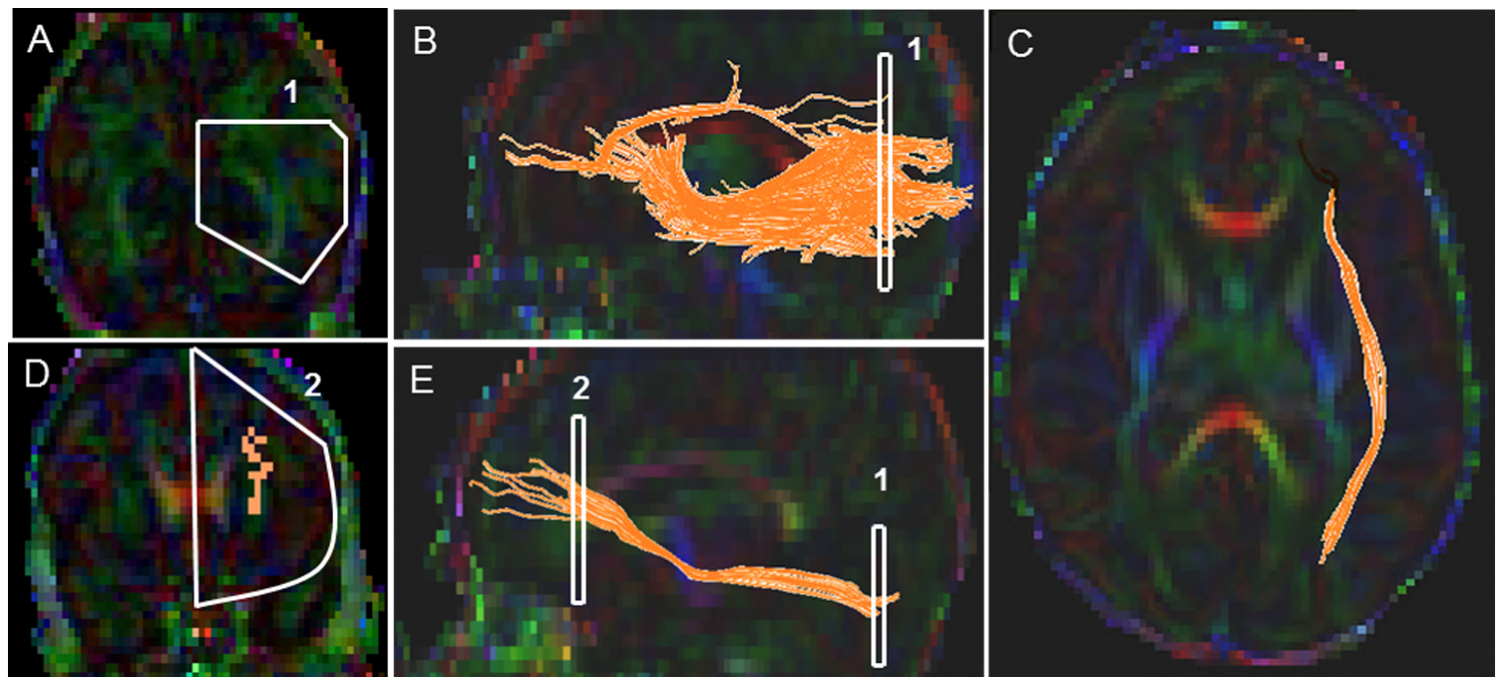
Location of ROIs on DTI color maps for the inferior fronto-occipital (IFO) tract in a preterm infant. (A) First polygonal shaped ROI in coronal view; (B) Fiber trajectory after the first ROI was drawn in sagittal view; (C) Final trajectory of the IFO tract in axial view; (D) Polygonal shaped second ROI in coronal view; (E) Final trajectory of the IFO tract and the starting and the ending ROI points of the tract in sagittal view.

Starting FA threshold of 0.11, stopping threshold of 0.05, and turning angle of 41 degrees were taken. For the first ROI, a coronal slice was identified at the level of the mid-point between the posterior edge of the parieto-occipital sulcus (POS) and the posterior edge of the cingulum (CG). Note that the POS, also known as the inter-hemispheric fissure, is a deep fissure located on the medial surface of the cerebral cortex, making the border between the parietal lobe and the cuneus of the occipital lobe. The POS was clearly identified on the FA map and Trace map images on the 2D sagittal images as a deep dark fissure located between the parietal lobe and the occipital lobe. The first coronal slice was selected by identifying the mid-way slice between the beginning and the end of the inter-hemispheric fissure (parietal occipital sulcus).Note that if the beginning and end of POS sulcus results in an odd number of slices, then the later of the two coronal slices was selected. The first ROI was drawn using the polygonal shape tool on the corresponding coronal slice as a pentagon, with the middle segment corresponding to the inter-hemispheric line, the top boundary covering the top part of the hemisphere, then arching down following the parallel edge of the left hemisphere and then back up obliquely meeting the middle inter-hemispheric line (Fig. 4A). Next the second ROI was located using first the axial slice marker which was placed such that it ran through the center of the body of the corpus callosum. Then the anterior most coronal slice was identified in which the genu of corpus callosum (intense red color fibers) just began to appear. The second ROI was drawn on the corresponding coronal slice using the “AND” function and the polygonal shape tool, and the entire left hemisphere was delineated (Fig. 4D). Last, non-IFO extraneous fibers (e.g. ILF, transverse pontine fibers, CST, cingulum) were then removed according to our predefined exclusion protocol (see Supplementary Appendix for details).

#### Uncinate Fasciculus (UNC) (Fig. 5)

**Figure 5:**
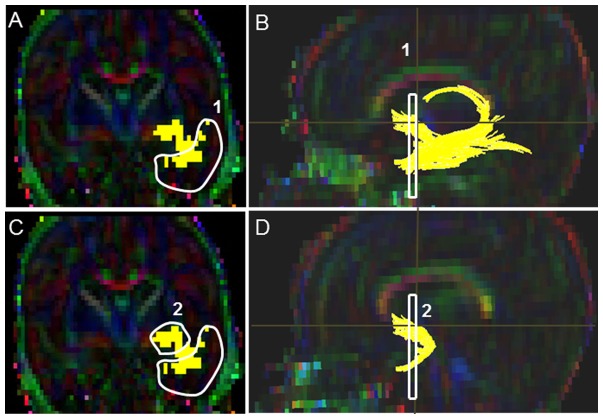
Location of ROIs on DTI color maps for the uncinate (UNC) tract in a preterm infant. (A) First polygonal shaped ROI drawn for segmenting the UCN in coronal view; (B) Fiber bundle after the first ROI was drawn in sagittal view; (C) The second ROI drawn anterior to the first ROI on the same coronal image; (D) Final trajectory of the UNC in sagittal view.

Starting FA threshold of 0.12, stopping threshold of 0.12, and turning angle of 60° were taken. First the mid-sagittal and axial slice markers were placed on the sagittal color map image such that the lateral sulcus, which separates the frontal and temporal lobes, was clearly seen. The first ROI was drawn by selecting the polygonal shape tool, approximately in the anterior one-third of the body of the corpus callosum which included the entire temporal lobe (Fig. 5A). The second ROI was drawn on the same slice, using the “AND” function and the polygonal shape tool in the inferior part of the frontal lobe on the same coronal slice as chosen for the first ROI (Fig. 5C). Last, the non-uncinate fibers were systematically removed according to our predefined exclusion protocol (see Supplementary Appendix for details).

#### Cingulum (CG), Cingulate Gyrus part (Fig. 6)

**Figure 6:**
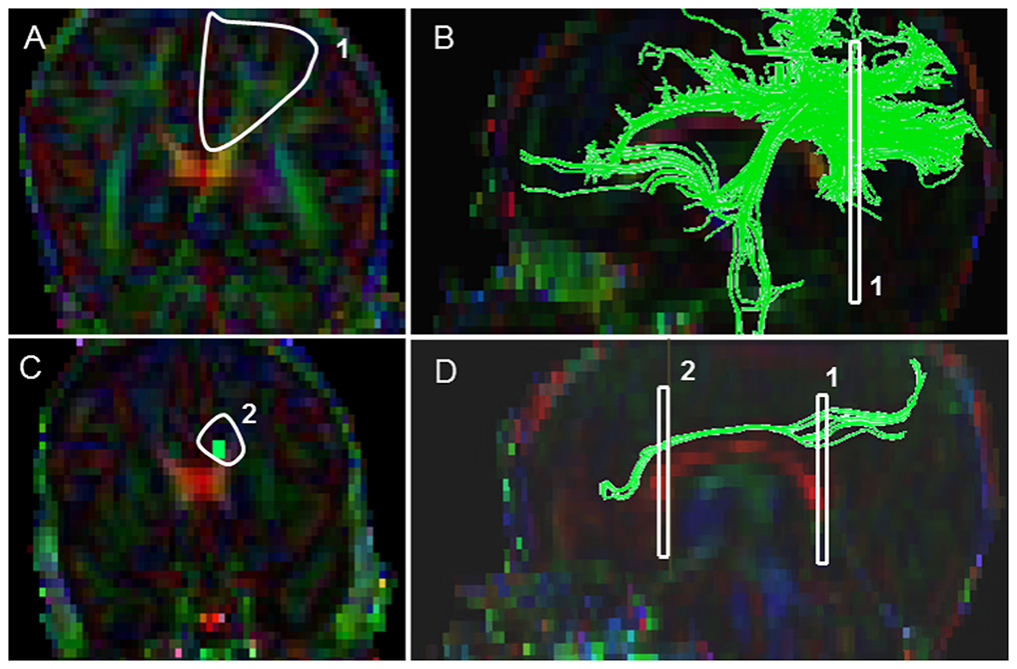
Location of ROIs on DTI color maps for the cingulum (CG) in a preterm infant. (A) First polygonal shaped ROI for segmenting the CG in the cingulate gyrus region in coronal view; (B) Fibers bundles after the placement of the first ROI sagittal view; (C) Second polygonal ROI in coronal view; (D) Final CG trajectory in sagittal view.

Starting FA threshold of 0.05, stopping threshold of 0.05, and turning angle of 70° were taken. In order to locate the first ROI, the sagittal slice in which the corpus callosum (CC) appeared most homogenous and symmetric in shaped was identified. Then on the corresponding coronal slice, the first ROI was drawn with the polygonal shape tool. The first ROI was drawn on the coronal slice starting from the top peripheral edge of the cortex and moved down perpendicularly to the top most part of the splenium of the corpus callosum (Fig. 6A). It was then extended to the outer edge of the cortex and then the top most part was arched back meeting the perpendicular edge along the periphery of the brain, forming an enclosed cone-shaped region. For the second ROI, the axial and coronal slice markers were adjusted such that they were both present at the level of the middle of the genu of the corpus callosum. Then on the corresponding coronal slice, the second ROI using the “AND” function with the polygonal shape tool was drawn as a heptagon (seven-sided polygon) around the prominent cluster of fiber voxels (Fig. 6C). Last, as per our pre-defined exclusion criteria, the non-cingulum extraneous fibers were systematically removed (see Supplementary Appendix for details).

#### Fornix (FX) (Fig. 7)

**Figure 7:**
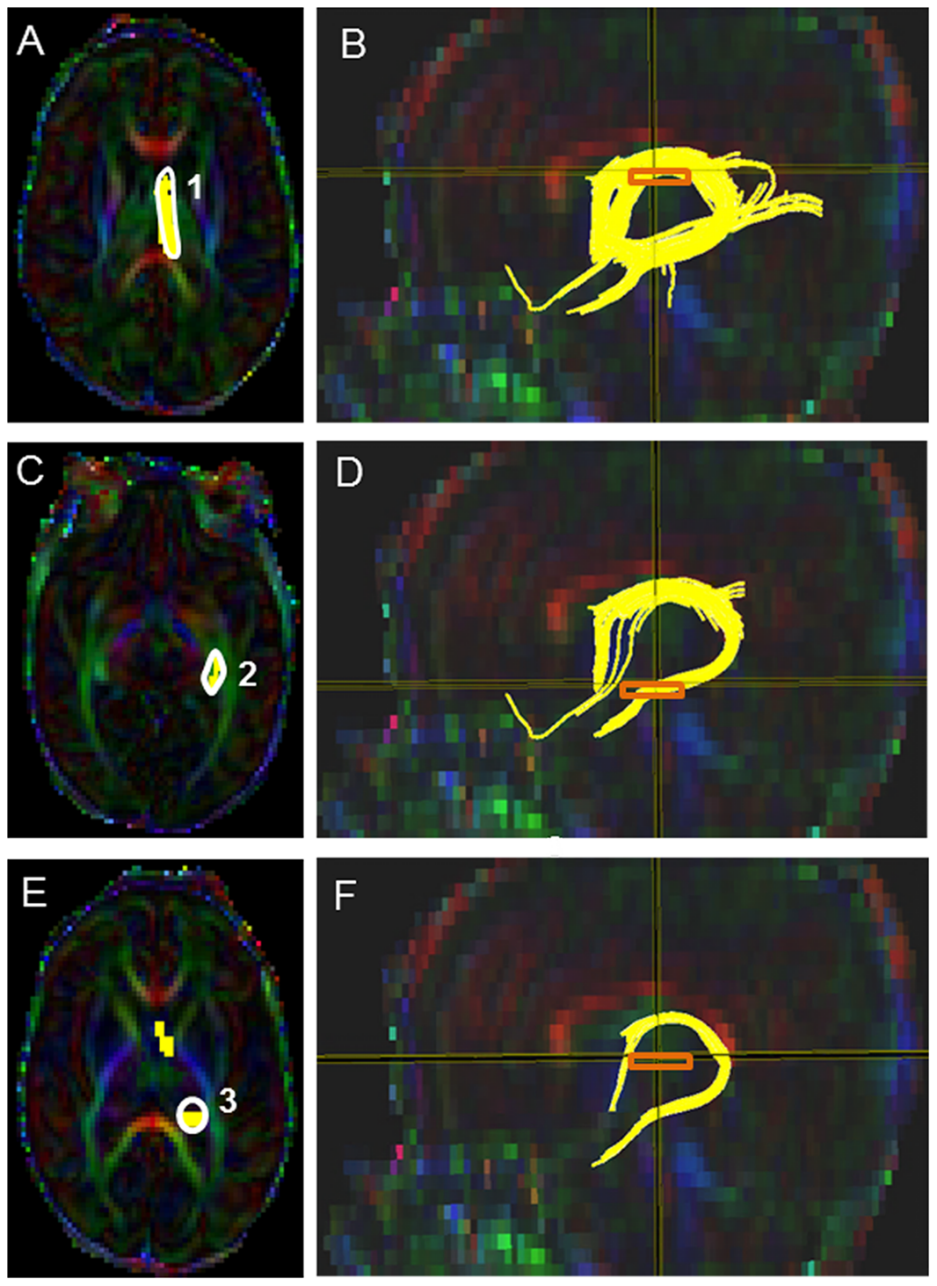
Location of ROIs on DTI color maps for the fornix (FX) in a preterm infant. (A) First polygonal shaped seed point of the first ROI around the FX in axial view; (B) Location of the fornix after the first ROI was drawn in sagittal orientation; (C) Second polygonal shaped ROI in axial view; (D) FX after the second ROI was drawn in sagittal view; (E) Third oval shaped ROI placement in axial view; (F) Final FX trajectory in sagittal view.

Starting FA threshold of 0.12, stopping threshold of 0.12, and turning angle of 60° were taken. For the first ROI, the first axial slice where the fornix first arises (appear as ‘N’ shaped intense green fibers) was identified. The first polygonal shaped ROI was drawn around the “N-shaped” fornix fiber bundle (Fig. 7A). Then the second tight polygonal shaped ROI was drawn using the ‘AND’ function around the intense green structures at the level near the hippocampus (Fig. 7C). The third ROI was selected in the slice in which the splenium and genu of the corpus callosum bundle appeared homogenous and in which the posterior limb of the internal capsule appeared as an intense blue structure. The “AND” function and an oval shape tool of 6.0 mm height and 6.0 mm width was used to draw the third ROI by placing the oval around the intense red region located over the splenium of the corpus callosum bundle (Fig. 7E). The non-fornix extraneous fibers (e.g. SCP, CC, CST, cingulum) were systemically removed according to our pre-defined exclusion criteria to achieve the final fornix tract (see Supplementary Appendix for details).

#### Optic Radiations (OR) (Fig. 8)

**Figure 8:**
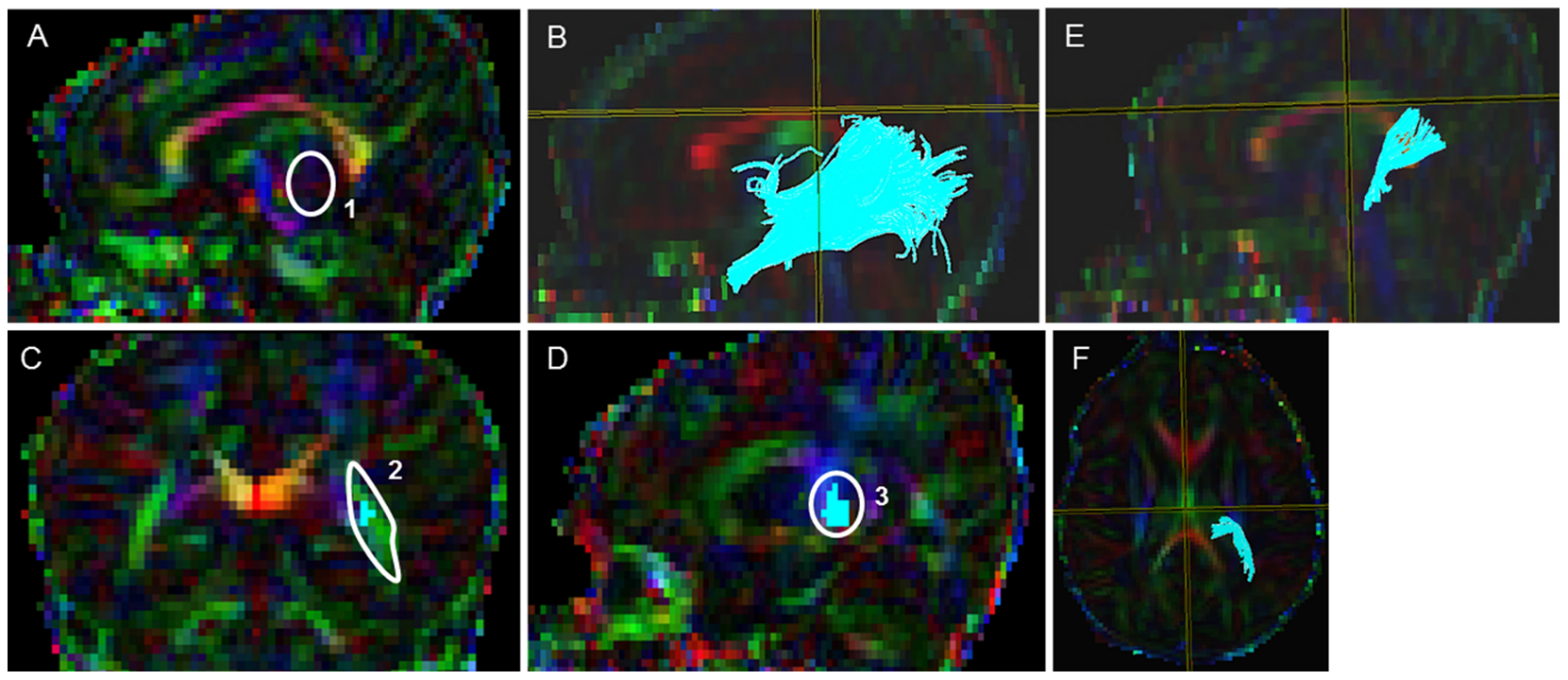
Location of ROIs on DTI color maps for the optic radiation (OR) in a preterm infant brain. (A) First ROI over the lateral geniculate (LGN) region in sagittal view; (B) Fibers after the placement of the first ROI in sagittal view; (C) Second polygonal shaped ROI around the sagittal stratum fibers in coronal view; (D) Third oval shaped ROI drawn at the anterior edge of the occipital lobe in sagittal view; (E&F) 3D Sagittal and axial images showing the final trajectory of the OR tract.

Starting FA threshold of 0.10, stopping threshold of 0.10, and turning angle of 70° were taken. For the first ROI, using the sagittal color map image, the axial slice marker was placed through the middle of the body of the corpus callosum (CC) and the coronal slice marker was positioned through the middle of the splenium of the corpus callosum. The sagittal slice in which the inverted ‘S-shaped’ curve and the lateral geniculate nucleus (LGN) fibers with deep intense red fibers first began to appear was selected. Then the middle of the LGN area was identified on the sagittal image. Following this, the middle of this intense red sphere on the sagittal slice was located, the corresponding coronal slice was located and the first ROI seed point using an oval of 8 mm width and 8 mm height was drawn with the ‘OR’ function, making certain that the anterior edge of the green voxels on the coronal slice around the intense red LGN area was highlighted (Fig. 8A). Then for location of the second ROI, the fiber voxels on the 2D image were turned off, the axial slice marker was centered through the body of the CC and the coronal maker was positioned at the center of the splenium of the CC. Then the second ROI was drawn using the tight poly tool and ‘AND’ function on the corresponding coronal slice demarcating the green sagittal stratum fibers. For the third ROI, the axial slice marker was positioned running through the body of the CC and the coronal slice marker was moved through the middle of the splenium of the corpus callosum (Fig. 8C). Then the third ROI was drawn with the oval of 8 mm width and 8mm height using the ‘AND’ function at the anterior side of the coronal markers where the intense blue-green threshold was noticed. Note: The ROI was placed in the anterior side and right over the intense blue-green threshold where the voxel clusters joins together on the 2D sagittal slice (Fig. 8D). Last, the non-OR extraneous fibers, which included SCP, transverse pontine fibers, CST, and cingulum were systematically removed as per our predefined exclusion protocol (see Supplementary Appendix for details).

#### Middle Cerebellar Peduncle (MCP) (Fig. 9)

**Figure 9:**
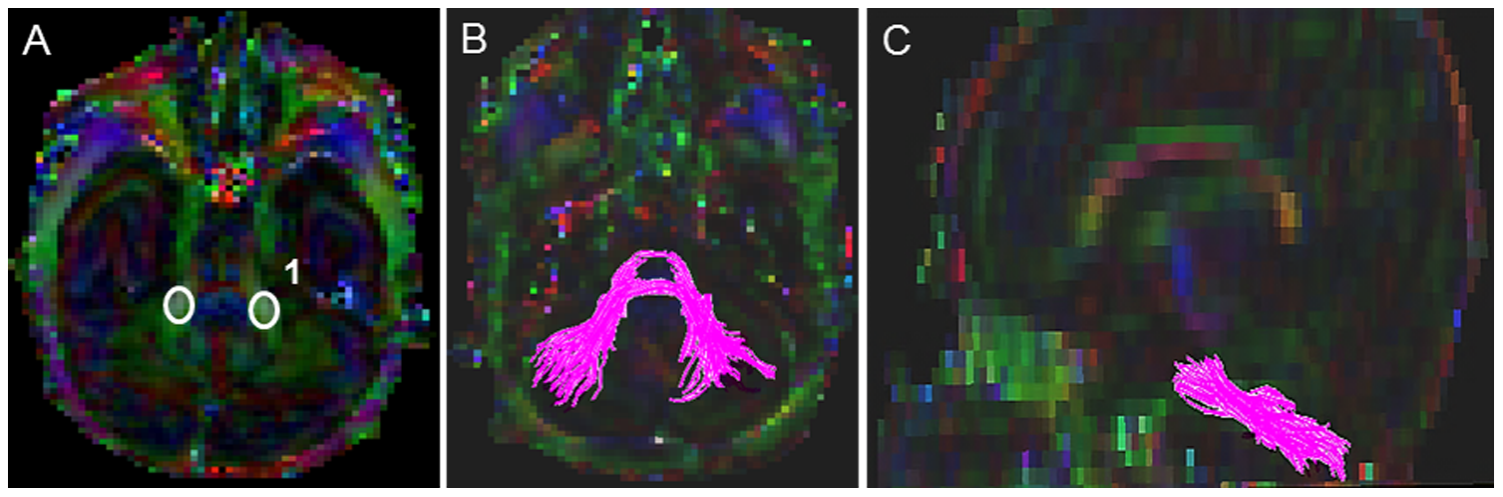
Location of ROIs on DTI color maps for the middle cerebellar peduncle (MCP) fiber bundle in a preterm infant brain. (A) Oval shaped ROI placed over the MCP tract in both lobes in axial view; (B&C) Complete trajectory of the MCP tract in axial and sagittal views.

Starting FA threshold of 0.13, stopping threshold of 0.05, and turning angle of 41° were taken. Note that the level of MCP slice selected for the ROI placement was usually seen 4–5 slices inferior to the level of superior cerebellar peduncle (SCP). Using the color map image, a single fixed oval of 4 mm width and 5 mm height was selected, and then two oval ROIs were placed near the middle of the most intense green MCP fiber bundle where there was a slight bulge present on both sides of the brain on the same axial slice (Fig. 9A). Both midline ROIs were placed approximately at same level on the axial slice. Last, according to our pre-defined exclusion protocol, the non-MCP extraneous fibers (e.g. SCP, transverse pontine fibers, CST, cingulum) were systematically removed (see Supplementary Appendix for details).

#### Superior Cerebellar Peduncles (SCP) (Fig.10)

**Figure 10:**
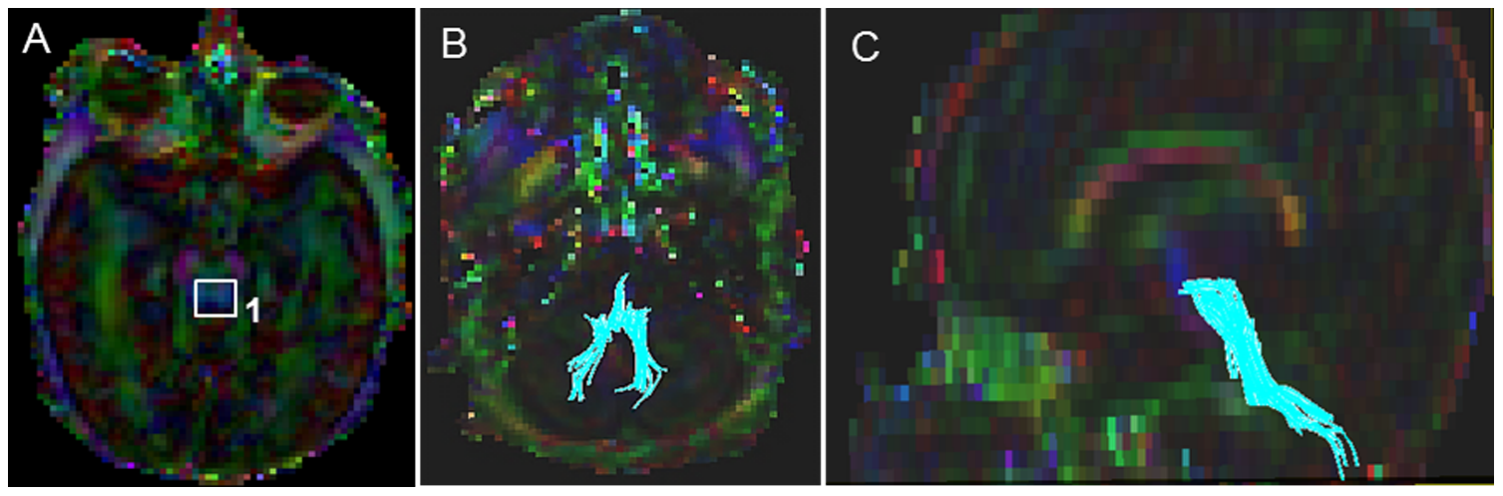
Location of ROIs on DTI color maps for the superior cerebellar peduncle (SCP) fiber bundle in a preterm infant brain. (A) Rectangular shaped ROI placed over the SCP tract in axial view; (B&C) Complete trajectory of the SCP fiber bundle in axial and sagittal views.

Starting FA threshold of 0.14, stopping threshold of 0.05, and turning angle of 41° were taken. A single ROI placement was required to complete the tractography for the SCP fiber bundle. First the axial slice in which the SCP first appeared as intense light blue structures with a red color dot (appeared in few full-term cases) was located. Note that the SCP fibers were seen decussating, forming a cross at the top, as seen on the axial slice and their posterior branch (one on each side) going to the cerebellum. These fibers are anatomically located posterior to the cerebral peduncles (the structures through which the corticospinal tract traverses its path). Then the single ROI of a fixed rectangular shape tool with 8.0 mm width and 8.0 mm height was selected, and was placed over the intense blue region (Fig. 10A). The non-SCP extraneous fibers (e.g. MCP, transverse pontine fibers, CST, cingulum) were systematically removed using our pre-defined exclusion protocol (see Supplementary Appendix for details).

### Statistical Analysis

Reliability, measurement error, and repeatability were assessed using intra-class correlation coefficients (ICC), Dice similarity index, within-subject standard deviation (SD), and repeatability coefficient. Following a rigorous training period, two trained raters independently segmented 29 ELBW and 15 full-term infants' DTI scans using a standardized protocol. In order to assess the intra- and inter-rater reliability and repeatability, after a two-week delay, both raters repeated segmentations for the same 44 cases. To assess measurement error, the within-subject SD was calculated by taking the square root of the mean within-subject variance. The latter was calculated by taking the average of the squared difference between two measurements, divided by two for all 44 subjects [34], [44]. The repeatability coefficient is defined as the variation in repeated measurements made on the same study samples taken under identical conditions. It is calculated as 2.77 (√2×1.96) times the within-subject SD. For the same subject, the difference between two measurements is expected to be less than 2.77 times the within-subject SD for 95% of pairs of observations [44], [45]. Repeatability is reported to be more clinically meaningful as a measurement difference that exceeds this value is more representative of a true clinical change and is unlikely to result from measurement error [45]. The similarity index is based on the spatial overlap between the two sets of segmentations for both *intra-* and *inter*-rater reliability measures. The ICC and Dice similarity index provide measures of reliability by correlating two measurements made on the same subject and are useful for assessing both *intra*-rater and *inter*-rater reliability for our three DTI measures. Because we included 29 training cases in the final test dataset of 44 subjects, we repeated the above measurement error and reliability statistics for the 15 preterm infants that were not part of the training dataset.

Using the R package (version 2.14.0), the *ICCest* function was used to calculate the ICC estimates, which are based on mean squares obtained by applying analysis of variance models to the data. It also estimates the confidence intervals using the variance components. We calculated the within-subject SD and repeatability coefficients using Microsoft Excel (2010 version) [45] and the Dice similarity index [46] using in-house MATLAB based program. While controlling for the effects of postmenstrual age at MRI scan, we used analysis of variance (ANOVA) to assess group differences in FA, MD, AD, RD, and total number of voxels and fibers between the ELBW preterm and term control infants. We also tested correlations between gestational age at birth and our six DTI metrics using linear regression. Stata IC/12 (Stata Corp, College Station, TX) was used for the ANOVA and regression analyses.

## Results

The mean gestational age and birth weight of the 29 ELBW infants was 25.2 (1.7) weeks and 771.8 (139.0) grams, respectively and postmenstrual age at MRI scan was of 38.4 (2.3) weeks. The 15 healthy term infants ranged from 37 to 41 weeks gestational age. Their mean (SD) birth weight was of 3177.2 (381.8) grams and postmenstrual age at MRI scan was of 39.1 (1.0) weeks. For six of our ten tracts, we had to exclude several ELBW subjects because their tract trajectories could not be outlined possibly due to pathology or regional signal inhomogeneity (signal drop out) arising from motion artifacts and image distortions. For the CST tract, three subjects were excluded; for the OR, two subjects; fornix, six subjects; ILF, nine subjects; CG, eight subjects; and for the IFO, 15 of the 44 subjects were excluded. In the healthy term infants, five tracts – IFO, ILF, CST, CG and OR – could not be segmented in all 15 subjects primarily due to regional signal drop outs. For the CST, one subject image could not be segmented and therefore excluded; for the ILF, two subjects; IFO, six subjects; CG in eight subjects; and OR could not be segmented in 4 of the 15 term subjects (see Supplementary Appendix for representative images).

The mean (SD) number of tract voxels, fiber numbers, and FA values for the 10 segmented tracts are presented in Tables 1, 2, and 3, respectively. The *intra*-rater reliability (within-subject SD, ICC, and Dice similarity index) and repeatability measures for these same measures are also presented in the same tables. The within-subject SD was within 1–2% and repeatability within 3–7% of the mean values for all 10 tracts. Tables 4, 5, and 6 present the inter-rater reliability and repeatability of the voxel numbers, fiber numbers, and the mean FA values between our two raters. Table 7 presents the intra-rater and inter-rater Dice similarity index to represent the degree of voxel overlap/similarity between the two iterations. Data for the 15 ELBW preterm infants that were not part of the initial training dataset are presented in Tables S1–S6 in the Supplementary Appendix. Measurement error, reliability, and repeatability measurements were the same or better than for the entire group of 44 infants.

**Table 1:** Mean total number of voxels with corresponding *intra*-rater measurement error, repeatability coefficient, and reliability data for ten white matter tracts in preterm and term infants.

Tract	Mean (SD)	With-subject SD	Repeatability	ICC (95% Cl)
MCP	1194.2 (350.4)	13.5	37.3	0.998 (0.989, 0.999)
SCP	398.9 (118.8)	6.2	17.2	0.996 (0.995, 0.999)
CC	3803.4 (1153.4)	18.6	51.5	0.995 (0.993, 0.998)
FX	233.1 (77.5)	5.4	14.8	0.978 (0.967, 0.992)
CG	353.4 (271.9)	5.6	15.4	0.985 (0.980, 0.989)
CST	180.3 (71.0)	1.8	5.1	0.997 (0.994, 0.999)
OR	281.9 (98.8)	3.9	10.9	0.981 (0.971, 0.996)
UNC	226.7 (108.7)	3.8	10.4	0.997 (0.996, 0.998)
IFO	564.9 (365.5)	4.3	11.9	0.988 (0.989, 0.999)
ILF	626.2 (345.3)	16.3	45.3	0.998 (0.997, 0.999)

**Table 2:** Mean total number of fibers with corresponding *intra*-rater measurement error, repeatability coefficient, and reliability data for ten white matter tracts in preterm and term infants.

Tract	Mean (SD)	With-subject SD	Repeatability	ICC (95% Cl)
MCP	311.2 (96.5)	4.3	11.9	0.997 (0.993, 0.998)
SCP	109.2 (39.6)	2.1	5.7	0.996 (0.993, 0.999)
CC	1468.1 (424.6)	37.2	103.0	0.994 (0.992, 0.999)
FX	28.2 (13.3)	0.6	2.5	0.995 (0.992, 0.998)
CG	42.2 (36.5)	0.9	2.6	0.995 (0.998, 0.999)
CST	16.5 (11.7)	0.4	1.1	0.998 (0.994, 0.999)
OR	54.8 (35.6)	1.3	3.6	0.998 (0.997, 0.999)
UNC	73.0 (39.0)	1.6	4.4	0.998 (0.994, 0.999)
IFO	58.9 (60.5)	0.7	2.0	0.998 (0.997, 0.999)
ILF	114.5 (84.9)	2.8	7.7	0.989 (0.997, 0.999)

**Table 3:** Mean fractional anisotropy with corresponding *intra*-rater measurement error, repeatability coefficient, and reliability data for ten white matter tracts in preterm and term infants.

Tract	Mean (SD)	With-subject SD	Repeatability	ICC (95% Cl)
MCP	0.24 (0.03)	0.01	0.03	0.988 (0.978, 0.994)
SCP	0.20 (0.02)	0.01	0.02	0.978 (0.968, 0.989)
CC	0.26 (0.03)	0.01	0.04	0.964 (0.959, 0.982)
FX	0.26 (0.04)	0.01	0.02	0.986 (0.977, 0.995)
CG	0.18 (0.03)	0.01	0.02	0.987 (0.973, 0.996)
CST	0.28 (0.05)	0.02	0.06	0.985 (0.973, 0.993)
OR	0.25 (0.03)	0.02	0.05	0.981 (0.967, 0.991)
UNC	0.23 (0.03)	0.02	0.06	0.971 (0.969, 0.982)
IFO	0.20 (0.02)	0.01	0.03	0.988 (0.996, 0.999)
ILF	0.16 (0.03)	0.01	0.04	0.985 (0.989, 0.998)

**Table 4:** *Inter*-rater measurement error, repeatability coefficient, and reliability data for total number of voxels of ten white matter tracts in preterm and term infants.

Tract	With-subject SD	Repeatability	ICC (95% Cl)
MCP	11.0	29.3	0.998 (0.988, 0.999)
SCP	4.0	11.0	0.998 (0.997, 0.999)
CC	110.6	306.4	0.991 (0.983, 0.995)
FX	5.1	14.2	0.998 (0.996, 0.999)
CG	7.7	21.5	0.995 (0.998, 0.999)
CST	4.0	11.1	0.997 (0.994, 0.998)
OR	10.8	29.9	0.988 (0.979, 0.994)
UNC	5.7	15.7	0.997 (0.995, 0.999)
IFO	15.1	42.0	0.998 (0.996, 0.999)
ILF	11.2	31.1	0.999 (0.998, 0.999)

**Table 5:** *Inter*-rater measurement error, repeatability coefficient, and reliability data for total number of fibers of ten white matter tracts in preterm and term infants.

Tract	With-subject SD	Repeatability	ICC (95% Cl)
MCP	6.5	18.0	0.996 (0.992, 0.998)
SCP	2.9	8.0	0.995 (0.991, 0.997)
CC	31.0	86.0	0.995 (0.990, 0.997)
FX	1.6	4.5	0.996 (0.992, 0.998)
CG	2.0	5.3	0.998 (0.997, 0.999)
CST	0.7	1.9	0.997 (0.994, 0.998)
OR	2.5	7.0	0.995 (0.991, 0.997)
UNC	2.1	5.8	0.997 (0.994, 0.998)
IFO	2.1	5.9	0.998 (0.997, 0.999)
ILF	3.2	8.8	0.998 (0.997, 0.999)

**Table 6:** *Inter*-rater measurement error, repeatability coefficient, and reliability data for mean fractional anisotropy of ten white matter tracts in preterm and term infants.

Tract	With-subject SD	Repeatability	ICC (95% Cl)
MCP	0.01	0.03	0.987 (0.976, 0.993)
SCP	0.01	0.03	0.977 (0.959, 0.987)
CC	0.01	0.03	0.966 (0.939, 0.982)
FX	0.01	0.03	0.986 (0.973, 0.993)
CG	0.01	0.02	0.989 (0.976, 0.995)
CST	0.02	0.04	0.986 (0.974, 0.993)
OR	0.02	0.04	0.981 (0.965, 0.990)
UNC	0.02	0.05	0.982 (0.937, 0.999)
IFO	0.01	0.02	0.984 (0.962, 0.993)
ILF	0.01	0.03	0.994 (0.988, 0.997)

**Table 7:** *Intra*-rater and *inter*-rater Dice similarity index for total number of voxels of ten white matter tracts in preterm and term infants.

Tract	Intra-rater Dice index	Inter-rater Dice index
MCP	0.976 (0.967, 0.984)	0.833 (0.808, 0.858)
SCP	0.980 (0.970, 0.990)	0.872 (0.848, 0.900)
CC	0.971 (0.956, 0.987)	0.829 (0.809, 0.849)
FX	0.985 (0.977, 0.993)	0.905 (0.876, 0.934)
CG	0.985 (0.977, 0.994)	0.877 (0.839, 0.914)
CST	0.980 (0.972, 0.988)	0.891 (0.854, 0.929)
OR	0.973 (0.962, 0.984)	0.829 (0.790, 0.866)
UNC	0.989 (0.985, 0.992)	0.895 (0.871, 0.919)
IFO	0.994 (0.992, 0.996)	0.900 (0.851, 0.950)
ILF	0.985 (0.978, 0.992)	0.816 (0.791, 0.841)

**Figure 11:**
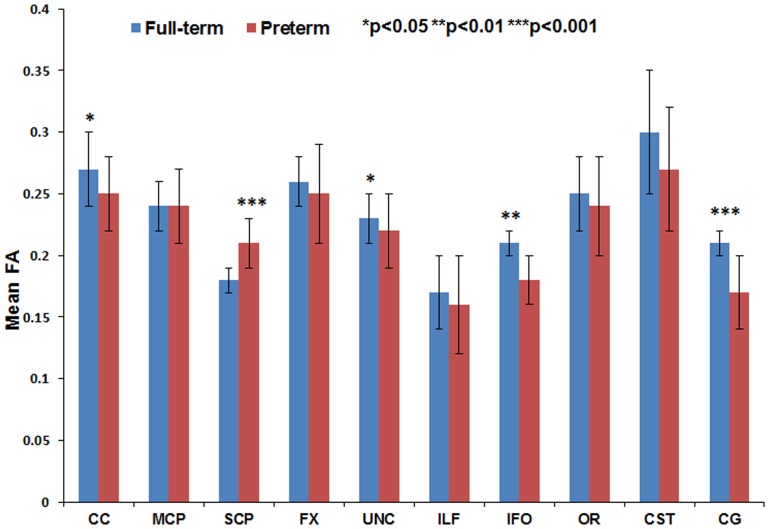
Mean fractional anisotropy for ten major white matter tracts in healthy term controls (blue bars) and ELBW infants (red bars).

Figures 11 through 16 present the mean (SD) FA, MD, RD, AD, number of fibers, and number of voxels for our preterm (red) and full-term control (blue) groups, respectively. For the vast majority of tracts, preterm infants exhibited lower FA, higher MD, higher AD, higher RD, and fewer fiber numbers and voxels than healthy term infants. Preterm infants exhibited significantly lower FA in the CC, UNC, IFO, and CG tracts and significantly higher MD in the CC, fornix, ILF, CST, and CG tracts as compared to term controls. The largest difference in fiber numbers and volume (i.e. voxel numbers) was observed in the CC, IFO, and CG.

**Figure 12:**
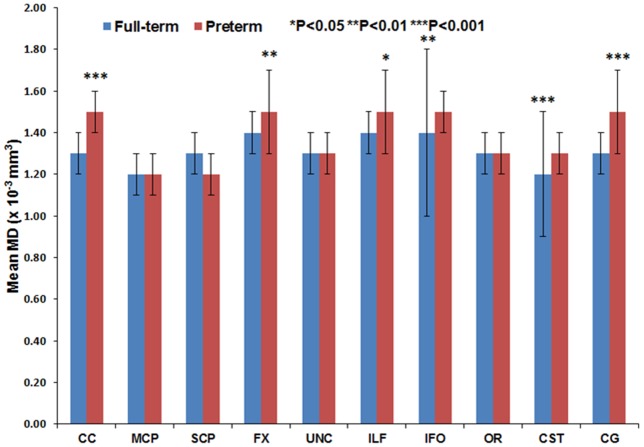
Mean MD for ten major white matter tracts in healthy term controls (blue bars) and ELBW infants (red bars).

**Figure 13:**
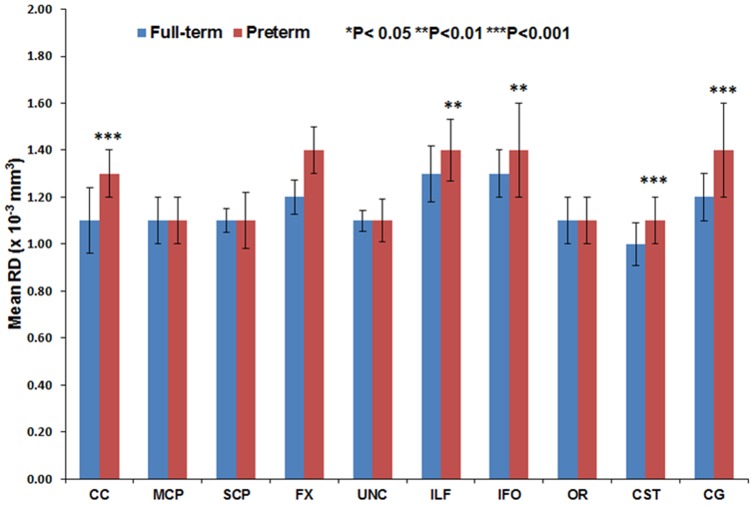
Mean RD for ten major white matter tracts in healthy term controls (blue bars) and ELBW infants (red bars).

**Figure 14:**
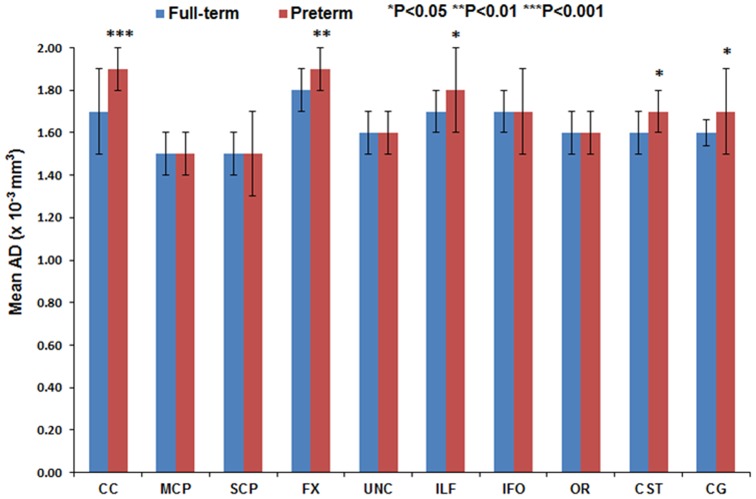
Mean AD for ten major white matter tracts in healthy term controls (blue bars) and ELBW infants (red bars).

**Figure 15:**
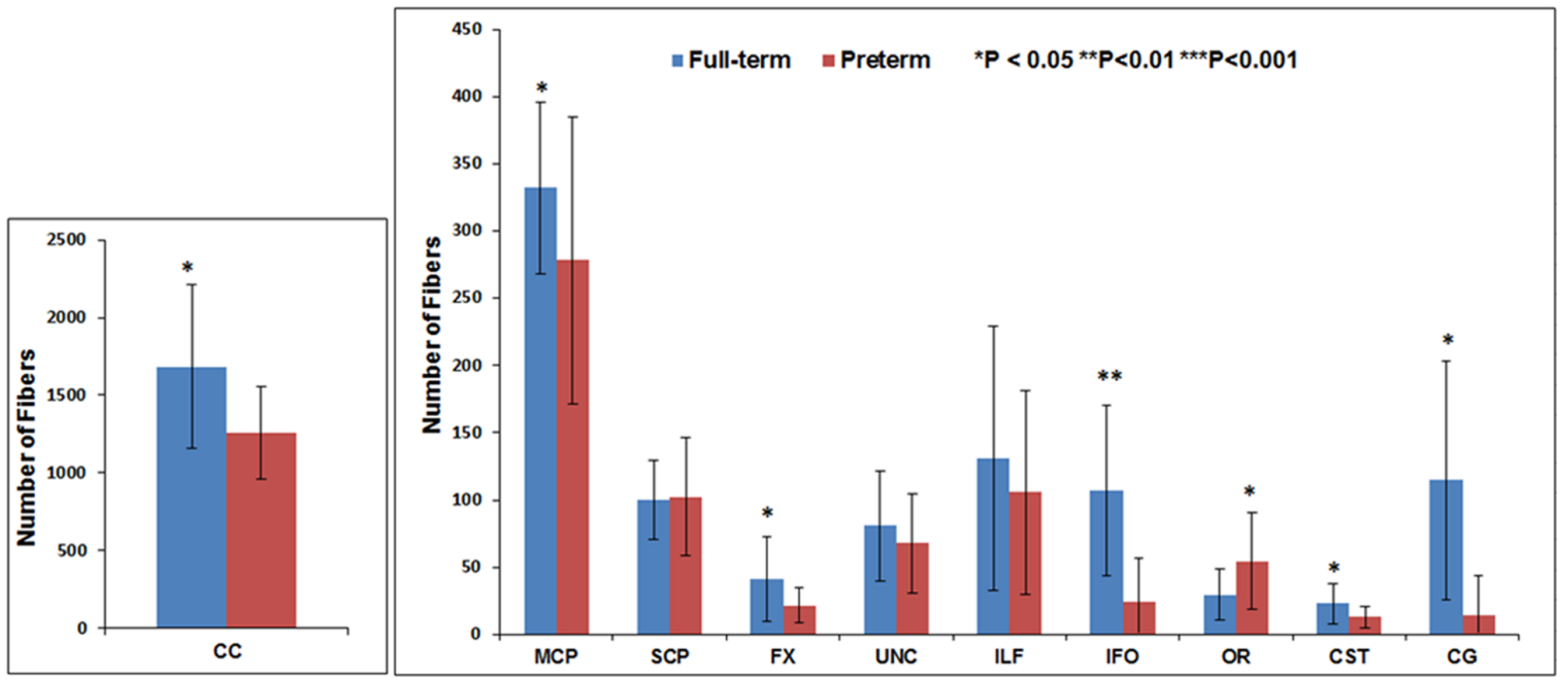
Mean number of fibers for ten major white matter tracts in healthy term controls (blue bars) and ELBW infants (red bars).

**Figure 16:**
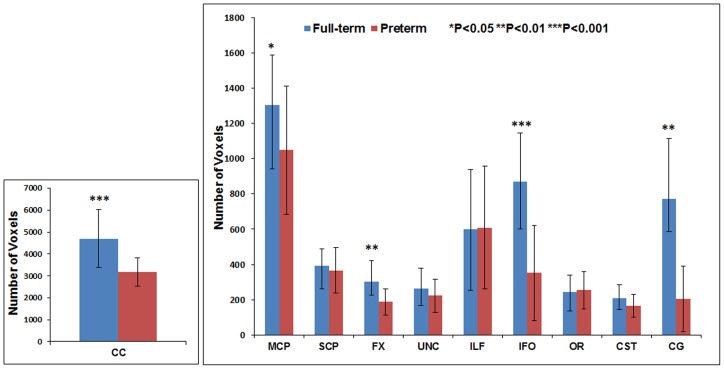
Mean number of voxels for ten major white matter tracts in healthy term controls (blue bars) and ELBW infants (red bars).

For six of the tracts – CC, FX, CG, IFO, ILF, and CST – gestational age at birth was strongly and directly associated with FA, number of fibers and number of voxels, and inversely associated with MD, AD, and RD. For the UNC, OR, MCP, and SCP, only one to two of the six DTI metrics were significantly associated with gestational age.

## Discussion

In this study we present a comprehensive and highly reliable neonatal protocol for deterministic tractography for ten white matter tracts. We were also able to demonstrate high intra-rater and inter-rater repeatability after two raters repeated measurements in all 44 subjects. The intra-rater Dice index was excellent, with a range of 0.97 to 0.99 and as expected, the inter-rater Dice index was lower (range: 0.82 to 0.91), but still within a very good reliability range. These results support our primary objective of developing a comprehensive, reliable and reproducible approach to neonatal tractography, suitable for further use in large randomized clinical trials or population-based studies.

A few previous studies have described their approach to neonatal tractography in preterm infants [30], [47], [50]–[52]. However, only two of these studies reported intra-rater reliability statistics (ICC; limits of agreement) [49], [51], [52]. Assessing reliability with ICC alone, a dimensionless quantity, can make interpretation subjective and difficult [45]. The correlation between repeated measurements will depend on the variability between subjects and can be misleading when solely used to evaluate measurement error [44]. Therefore, Bland and Altman (1996) have argued for the need to report within-subject SD and repeatability coefficient, which are more accurate measures of measurement error and repeatability, respectively. The repeatability coefficient is defined as the 95% interval for change between two or more repeat measurements [44]. Our repeatability measures indicate that for studies comparing different groups (e.g. randomized clinical trial) or the same individual imaged over time, differences in tract measures greater than 3–7% can be detected by using our approach. This can result in dramatic reductions in sample size needs and total study costs. Our tractography protocol can also be applied to other high-risk neonatal groups, including newborns with stroke, encephalopathy, congenital anomalies, and congenital heart disease.

The group differences we observed between ELBW and healthy term infants in FA, MD, number of fibers and number of voxels also confirm the validity of our measures. As expected for many of the tracts, the full-term control group exhibited greater fiber numbers, fiber volume, and mean FA values and lower MD as compared to ELBW infants, with up to five tracts exhibiting significant differences. However, term infants exhibited fewer SCP fibers with lower FA and higher MD than ELBW infants. Because we excluded ELBW infants with severe brain injuries, we expected to find no difference. Our smaller group sizes may have resulted in some sampling bias.

The absolute mean values for FA and MD we observed were comparable to several prior reports. Using a similar deterministic tractography approach, several neonatal studies [47], [48], [51], [53]–[55] have reported the differential maturation of white matter bundles during early months of infant brain development. This study [54] reported the FA and MD measures in very preterm infants at term for the cingulum, fornix, and IFO bundles were in agreement with our data. Another study [48] reported mean FA of 0.30 for the optic radiation and 0.26 for the CST; our corresponding values were 0.25 and 0.28. Likewise, another study [55] MD values in the brainstem and cerebellum tracts agreed with our MD measures for the MCP and SCP tracts. However, compared to our findings, Thompson et al. [33] observed lower CC FA of 0.21 in term and 0.20 in preterm infants. These differences might be attributable to differences in tractography technique, ROI selection, and/or tract threshold values selected for the study.

No previous groups have reported the number of fibers or voxels in ELBW infants or compared preterm with healthy term infants. However, corpus callosum cross-sectional area using DTI color maps and volumes using T2-weighted scan [33], [37] were reported. Both studies showed marked reduction in CC area and volume, consistent with our findings of reduced number of voxels and fibers. The corpus callosum is the largest white matter fiber bundle and is crucial for inter-hemispheric communication of motor, sensory, and cognitive information. Another study reported injury and delayed development of the CC is associated with abnormal motor and cognitive outcomes at 2 years [56].

We also observed a large group differences in the number of fibers and voxels in the cingulum and IFO tracts. The cingulum is located immediately above the corpus callosum and is part of the limbic system. The importance of this structure in adult psychiatric and cognitive diseases has been widely studied [56]. However, its role in prematurity associated disabilities is still emerging [29]. The IFO is a large intra-hemispheric tract that connects the frontal, temporal, and occipital lobes and facilitates integration of auditory and visual association cortices with prefrontal cortex. Injury to the IFO might explain the poor language comprehension and semantic processing observed in very preterm infants [5].

The high reliability and repeatability metrics for ten major white matter tracts was a major strength of our study. This was facilitated by our use of an extensive training period and a multiple ROI approach that was based on neuroanatomical knowledge from adult studies. Marked group differences between preterm and term infants in tract micro and macrostructure provided further evidence that our approach is clinically useful. However, some limitations involved with tractography techniques deserve mention. The results are dependent upon the quality of the DTI image acquisition, including spatial resolution, signal to noise ratio, and motion artifacts and the image pre-processing algorithm used. Tractography measures such as fiber number are semi-quantitative at best, and have not been validated with postmortem studies. Nevertheless, they may offer another useful measure of tract integrity to study for individual patients over time or to compare different groups of subjects. Fiber crossing in tractography is also a crucial issue in determining the directionality of the major eigenvector. At the point of crossing fibers, lower than normal anisotropy is observed and directions of the actual eigenvector do not correspond to the directions of both fiber tracts [57], [58]. Employing the use of diffusion spectrum imaging and q-ball imaging that involve high spatial resolution should further increase sampling efficiency and improve signal to noise ratio. Future testing of probabilistic tractography may also account for the inherent limitations of deterministic tractography and provide more robust models of distributed connectivity. We experienced difficulty in segmentation of five tracts in full-term infants and six tracts in ELBW infants due to regional signal drop out, signal inhomogeneity and image noise. Most of this was likely a result of motion artifacts. We elected to use feeding and swaddling and noise protection to avoid the use of sedation in these healthy subjects [59]. Considering our strict exclusion criteria [37], it is highly unlikely these tracts could not be segmented as a result of brain injury. However, for some of our ELBW infants where full segmentation was not possible, this rationale is possible because these infants are known to exhibit high rates of brain injury/delayed development. We elected to use a more conservative analysis strategy by excluding these subjects. Inclusion of these high-risk subjects would have resulted in greater tract-specific maturational differences between term and preterm infants. In our study, tractography of certain tracts such as IFO and the cingulum fiber bundle were difficult to manually segment, possibly due to pathology and/or motion artifacts noticed in some of the preterm infant scans. Both of these tracts showed large differences in fiber number, voxel number, and diffusion metrics between preterm and term infants. Ultimately, some of these limitations can be overcome with automated neonatal tractography algorithms, as we are currently pursuing.

Diffusion measures such FA, and MD are now well accepted to represent white matter microstructural development. In extremely preterm infants, emerging studies suggest they are associated with perinatal interventions and neurodevelopmental impairments [60], [61], [57]. Use of full tract based measures of microstructure and macrostructure as compared to ROI-based measures should prove more robust in diagnosis of injury/delay and prediction of outcomes. Advanced MRI measures such as DTT are more sensitive than current neonatal imaging measures and when combined, could prove to be a valuable surrogate endpoint for neurodevelopmental outcomes. In particular, earlier knowledge of cognitive impairment risk, which cannot be accurately diagnosed until 5 years of age, holds the greatest potential for benefitting preterm infants [62].

## Conclusion

We demonstrated development of highly reliable and reproducible comprehensive methods for manual segmentation of important white matter tracts in ELBW and term neonates. Extremely preterm infants exhibited fewer fiber numbers and reduced volume and/or abnormal microstructure in a good majority of studied white matter tracts, such as CC, IFO, CG, CST, MCP, and FX, consistent with delayed white matter development and/or injury [28]–[30], [33], [37], [62]. This protocol could serve as a valuable tool for prompt evaluation of the impact of neuroprotective therapies and as a prognostic biomarker for neurodevelopmental impairments.

### Data Accessibility Statement

Any researcher wishing to access our data may do so by stating their research objective(s) and desire to access the data in an email to the corresponding author.

## Supporting Information

Figure S1 DTI color maps of the difficult to segment corticospinal tract (CST) in a representative healthy full-term infant. (A) Polygonal shaped first ROI drawn over the cerebral peduncle in axial view; (B) Tract after the first ROI was drawn in sagittal view; (C) Second ROI drawn over the pre-central gyrus in axial view; (D) the trajectory of the CST in sagittal view impacted by motion artifacts and signal inhomogeneity.
(TIF)

Figure S2 DTI color maps of the difficult to segment optic radiation (OR) in a representative healthy full-term infant brain. (A) First ROI over the lateral geniculate region in sagittal view; (B) Fibers after the placement of the first ROI in sagittal view; (C) Second polygonal shaped ROI around the sagittal stratum fibers in coronal view; (D) Third oval shaped ROI drawn at the anterior edge of the occipital lobe in sagittal view; (E&F) 3D Sagittal and axial images showing the final trajectory of the OR tract affected by motion artifacts and signal inhomogeneity.
(TIF)

Figure S3 DTI color maps of the difficult to segment cingulum (CG) in a representative healthy full-term infant. (A) First polygonal shaped ROI for segmenting the CG in the cingulate gyrus region in coronal view; (B) Fibers bundles after the placement of the first ROI sagittal view; (C) Second polygonal ROI in coronal view; (D) Final CG trajectory in sagittal view affected by motion artifacts and signal inhomogeneity.
(TIF)

Figure S4 DTI color maps of the difficult to segment inferior-longitudinal fasciculus (ILF) in a representative healthy full-term infant brain. (A) First ROI for segmenting the ILF in coronal view, covering the entire left hemisphere; (B) Fiber trajectory after the first ROI was drawn in sagittal view; (C) Polygonal shaped second ROI in coronal view; (D&E) Final trajectory of the ILF tract and the locations of the two ROIs in sagittal and axial views impacted by motion artifacts and signal inhomogeneity.
(TIF)

Figure S5 DTI color maps of the difficult to segment inferior fronto-occipital (IFO) in a representative healthy full-term infant brain. (A) First polygonal shaped ROI in coronal view; (B) Fiber trajectory after the first ROI was drawn in sagittal view; (C) Polygonal shaped second ROI in coronal view; (D) Starting and the ending ROI points of the tract in sagittal view affected by motion artifacts and signal inhomogeneity; (E) No fiber tract could be segmented.
(TIF)
