# Supplementary figures and images for: Reliability and Repeatability of Quantitative Tractography Methods for Mapping Structural White Matter Connectivity in Preterm and Term Infants at Term-Equivalent Age
Source: PLoS One. 2014 Jan 24;9(1):e85807. doi: 10.1371/journal.pone.0085807 (PMC3901659; doi:10.1371/journal.pone.0085807)

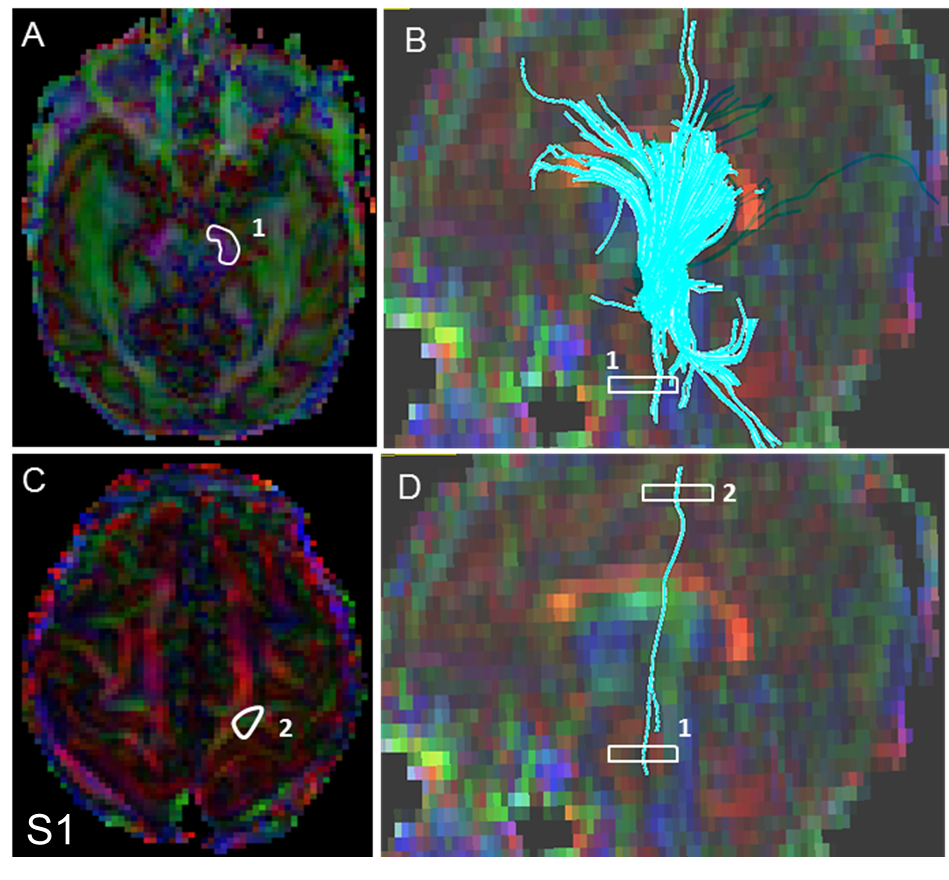

Supplement: Figure S1 — DTI color maps of the difficult to segment corticospinal tract (CST) in a representative healthy full-term infant. (A) Polygonal shaped first ROI drawn over the cerebral peduncle in axial view; (B) Tract after the first ROI was drawn in sagittal view; (C) Second ROI drawn over the pre-central gyrus in axial view; (D) the trajectory of the CST in sagittal view impacted by motion artifacts and signal inhomogeneity. (TIF) [file pone.0085807.s001.tif]

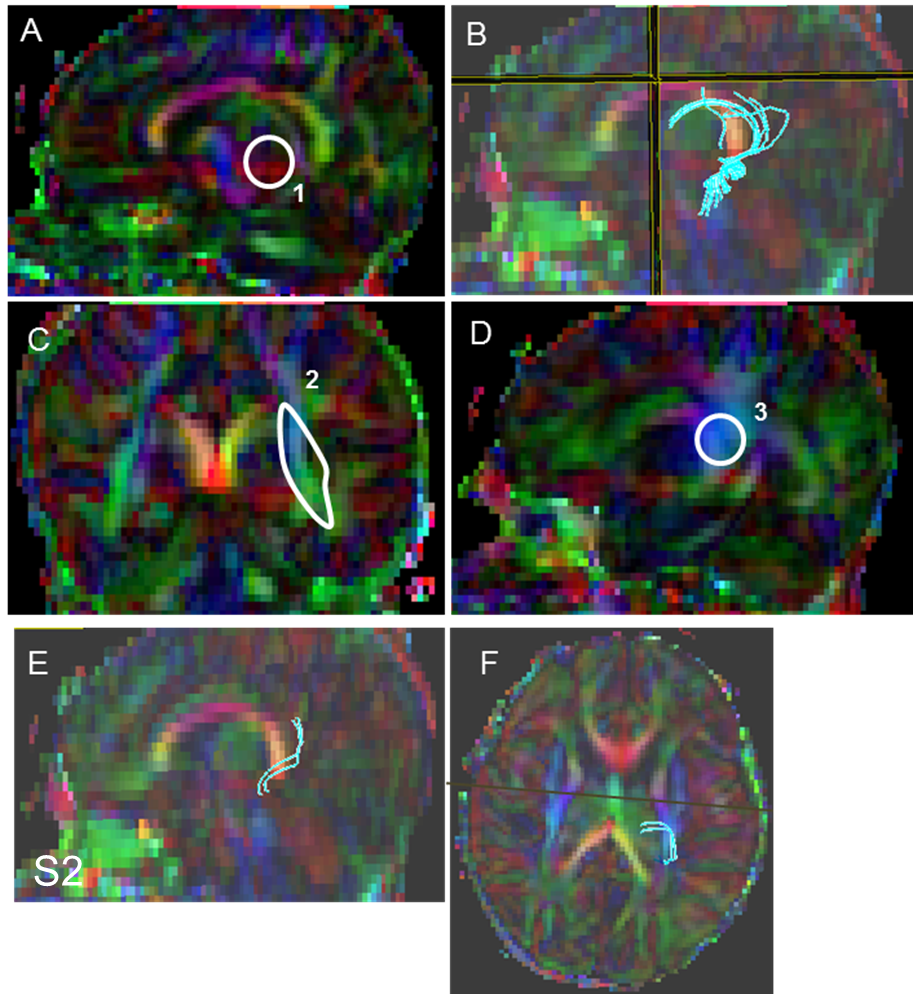

Supplement: Figure S2 — DTI color maps of the difficult to segment optic radiation (OR) in a representative healthy full-term infant brain. (A) First ROI over the lateral geniculate region in sagittal view; (B) Fibers after the placement of the first ROI in sagittal view; (C) Second polygonal shaped ROI around the sagittal stratum fibers in coronal view; (D) Third oval shaped ROI drawn at the anterior edge of the occipital lobe in sagittal view; (E&F) 3D Sagittal and axial images showing the final trajectory of the OR tract affected by motion artifacts and signal inhomogeneity. (TIF) [file pone.0085807.s002.tif]

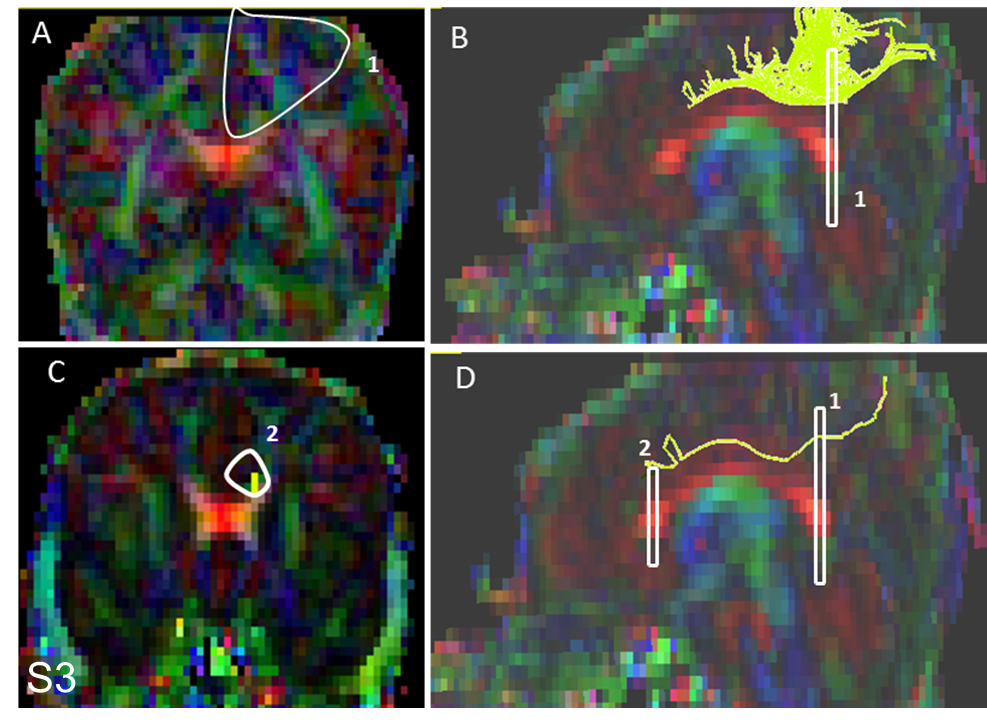

Supplement: Figure S3 — DTI color maps of the difficult to segment cingulum (CG) in a representative healthy full-term infant. (A) First polygonal shaped ROI for segmenting the CG in the cingulate gyrus region in coronal view; (B) Fibers bundles after the placement of the first ROI sagittal view; (C) Second polygonal ROI in coronal view; (D) Final CG trajectory in sagittal view affected by motion artifacts and signal inhomogeneity. (TIF) [file pone.0085807.s003.tif]

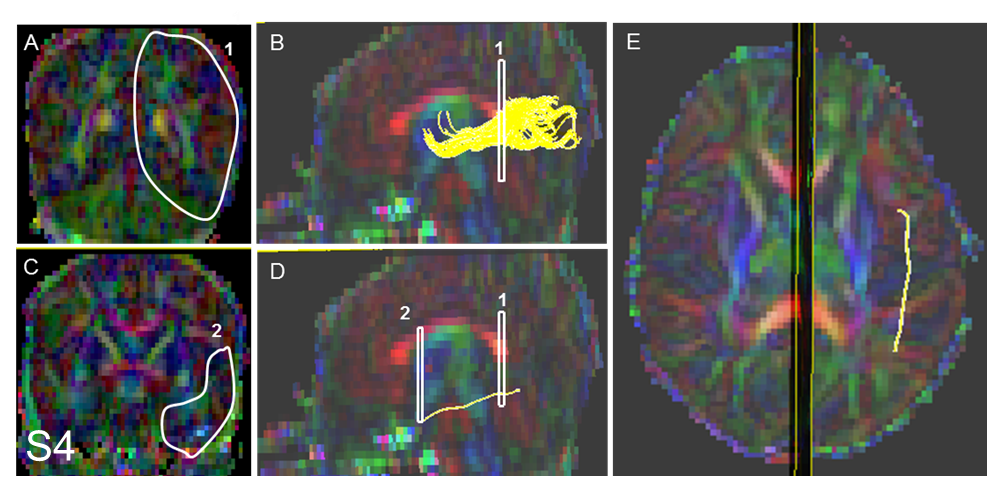

Supplement: Figure S4 — DTI color maps of the difficult to segment inferior-longitudinal fasciculus (ILF) in a representative healthy full-term infant brain. (A) First ROI for segmenting the ILF in coronal view, covering the entire left hemisphere; (B) Fiber trajectory after the first ROI was drawn in sagittal view; (C) Polygonal shaped second ROI in coronal view; (D&E) Final trajectory of the ILF tract and the locations of the two ROIs in sagittal and axial views impacted by motion artifacts and signal inhomogeneity. (TIF) [file pone.0085807.s004.tif]

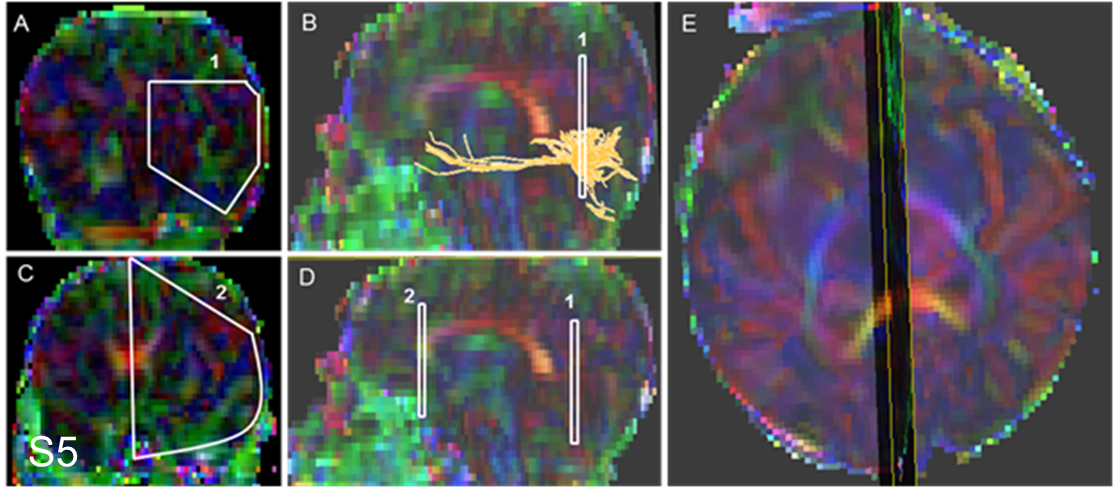

Supplement: Figure S5 — DTI color maps of the difficult to segment inferior fronto-occipital (IFO) in a representative healthy full-term infant brain. (A) First polygonal shaped ROI in coronal view; (B) Fiber trajectory after the first ROI was drawn in sagittal view; (C) Polygonal shaped second ROI in coronal view; (D) Starting and the ending ROI points of the tract in sagittal view affected by motion artifacts and signal inhomogeneity; (E) No fiber tract could be segmented. (TIF) [file pone.0085807.s005.tif]
